# Ectopic pheochromocytoma or paraganglioma of the ZUCKERKANDL organ: A case report and review of the literature

**DOI:** 10.1016/j.ijscr.2019.06.007

**Published:** 2019-06-12

**Authors:** Nayi Zongo, Adjirata Koama, Benilde Marie Ange Kambou/Tiemtoré, Nina A. Nde/Ouédraogo, Maurice Zida, Marie N.L. Ouédraogo, Edgard Ouangré, Adama Sanou, Olga Melanie Lompo, Ousséni Diallo, Claudine Lougué/Sorgho, Rabiou Cissé

**Affiliations:** a Visceral Surgery at Yalgado Ouedraogo University Hospital Centre (CHUYO), Burkina Faso; b Visceral Surgery at Blaise Compaoré National Hospital (HNBC), Burkina Faso; c Pathological Anatomy, CHUYO, Ouagadougou, Burkina Faso; d Souro Sanou University Hospital Centre of Bobo Dioulasso (CHU SS), Burkina Faso; e Radiology and Medical Imaging Department, University Hospital Centre (CHU) of Yalgado, Ouagadougou, Burkina Faso; f Radiology and Medical Imaging Department, University Hospital Centre (CHU) of Bogodogo, Ouagadougou, Burkina Faso

**Keywords:** Pheochromocytoma, Diagnosis, Surgery

## Abstract

**Introduction:**

Paraganglioma of the ZUCKERKANDL organ are rare. Diagnosis is based on clinical, radiological and biological arguments. We report a case to describe our surgical procedure and insist on the necessity of preoperative diagnosis.

**Presentation of case:**

BA, 52-years-old male patient was seen in consultation for left hypochondrium pains. The clinical examination had revealed a painful tumefaction in the left flank and the left hypochondrium. A deep mass was observed, but was difficult to be assessed, due to pain. Abdominal-pelvic CT scan with contrast injection had revealed a tissue mass, suggesting a tumor of the tail of the pancreas. Laparotomy showed this mass was not attached to the tail of the pancreas, and was along the abdominal aorta up to the aortic bifurcation. Upon touching the mass, blood pressure raised up to 240 mmHg. A least mobilization of the mass and the use of nicardipine helped maintain blood pressure below 180mmhg. Dissection was carried out from the aortic bifurcation to the TREITZ’s angle and the mass was removed. The follow-ups were characterized by low blood pressure a few minutes following the resection of the mass.

**Discussion:**

Pheochromocytoma is rare. The Clinical signs, Abdominal-pelvic CT scan and biology are the steps of the preoperative diagnosis. The surgery consists a lumpectomy. The resuscitation determines the patient’s prognosis.

**Conclusion:**

Pheochromocytoma is an unusual mass. Preoperative diagnosis can be difficult in pauci-symptomatic cases. One should consider this in the face of any abdominal mass, so as to improve planning of resuscitation which determines the patient’s prognosis.

## Highlights

• **EPIDEMIOLOGY**: paraganglioma of the ZUCKERKANDL organ is a rare neuroendocrine tumour but must be known.
• **DIAGNOSIS**: The typical presentation which is the Menard Triad of symptoms, associating headaches, sweating and palpitation. Preoperative diagnosis can be difficult in pauci-symptomatic cases. The Clinical signs, Abdominal-pelvic CT scan and biology are the steps of the preoperative diagnosis.
• **TREATMENT**: Treatment is surgical. Preoperative medical preparation is aimed at reducing risks of peroperative hemodynamic instability. The anesthetist should therefore prepare himself to manage blood pressure peaks during the tumour’s dissection, but also the possible low blood pressure at the end of exeresis. Surgery remains the key element of treatment and consists in exeresis of the paragaglioma.
• **PROGNOSIS**: paraganglioma of the ZUCKERKANDL organ is often a benign tumor. The resuscitation determines the patient’s prognosis.

## 1 Introduction

Pheochromocytoma is a rare neuroendocrine tumour [1,2]. It preferentially locates in the adrenal medulla [1,2]. It may locate in the extra-medulla and is then called paraganglioma (PGL) [1]. Intra-abdominal paraganglioma preferentially develops in the para-aortic ZUCKERKANDL organ, a lymph node at the root of the low mesenteric artery [1]. It is special because of the synthesis and secretion of hormones called catecholamines i.e. adrenaline, noradrenaline and dopamine [1]. These hormones cause a high adrenergic state which characterizes clinically by significant hemodynamic changes with terrible cardiovascular complications (high blood pressure, palpitations) and metabolic complications [1,3]. Suspected clinically, diagnosis was supported by biology, imaging and then confirmed by histology of the excised tissue [[1], [2], [3]]. Treatment is surgical and requires a careful medical preparation [3]. Throughout its evolution there may be recurrences, but the outcome is most often favourable [4]. Despite their scarcity, European western literature of pheochromocytomas is rich. However, in our country, there are very few publications on pheochromocytoma. We report a case of paraganglioma of the ZUCKERKANDL organ, with a view to describing our diagnostic adventures and patient’s care. The management of this case as well as literature search was performed and the work has been reported in line with the SCARE criteria [5].

## 2 Presentation of case

**Fig. 1:**
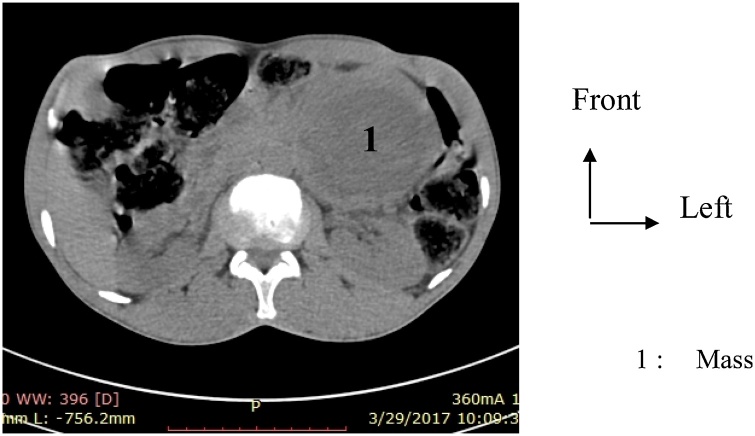
CT scan image mimicking a cancer of the tail of the pancreas.

The patient is 52-years-old and a shepherd living in Dori, was seen in cancer consultation on April 2017, for left hyponchondrium pains for the past 3 months. He was known to be hypertensive, but not diabetic. The clinical examination had revealed a painful tumefaction in the left flank and in the left hypochondrium. A deep mass could be observed, but was difficult to be assessed due to pain. Abdominal-pelvic CT scan with contrast injection had helped find an inhomogeneous tissue mass suggesting a tumour of the tail and body of the pancreas, measuring 86 × 83 mm (Fig. 1).

**Fig. 2:**
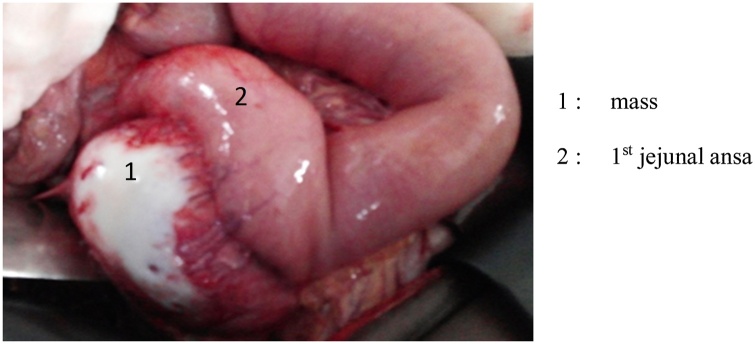
Mass in contact with the duodeno-jejunal angle (Treitz).

**Fig. 3:**
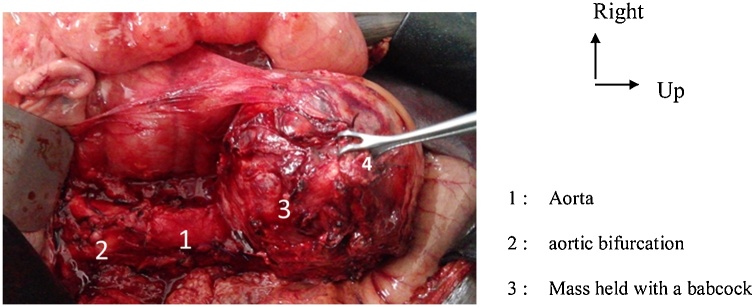
Dissection of the mass using Babcock’s pliers and with no hand contact, allowing the least mobilization possible.

**Fig. 4:**
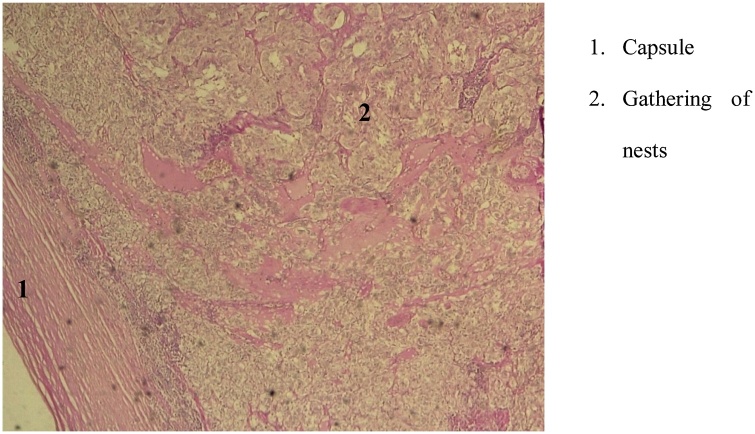
Microscopic image of pheochromocytoma at low magnification showing the cell architecture with a capsule limit and a gathering of cells into nests.

Preoperative workup revealed a sinus tachycardia. The indication for surgery was corporo-caudal spleno-pancreatectomy. Laparotomy showed a mass which was separated from of the tail of the pancreas, multicoloured, lying against the duodeno-jujenal angle through some small vessels, and along the abdominal aorta up to the aortic bifurcation (Fig. 2). Upon contact with the mass blood pressure rose up to 240 mm Hg. A minimal mobilization of the mass with a Babcock (Fig. 3) and the use of Nicardipine helped maintain blood pressure below 180 mm Hg. The dissection extended from the aortic bifurcation to the TREITZ’s angle, thus allowing removing the mass. The follow-ups were marked by low blood pressure a few minutes after resection of the mass. This was addressed with vascular replenishment. Forty-eight hours after surgery, the patient presented acute edema of the lungs, which was treated with diuretics. Histology confirmed the diagnosis of pheochromocytoma (Fig. 4). After a follow-up of 17 months, the patient did not make any complaint and his blood pressure was normal.

## 3 Discussion

Described for the first time by Félix Frankel in 1884, pheochromocytoma remains a rare tumour [6]. It secretes catecholamines whose vascular, cardiac or metabolic effects vary from one individual to another [[1], [2], [3]]. The ubiquity and diversity of catecholamine receptors are responsible for the clinical polymorphism of pheochromocytomas [2]. When it is not incidentally discovered during a morphological examination, pheochromocytoma can be at the origin of a pleomorphic symptomatology that is why it is qualified as “great imitator’’ [1]. In our case, the clinical manifestation was a known hypertension which was not enough to suspect the diagnosis preoperatively. The typical presentation which is the Menard Triad of symptoms, associating headaches, sweating and palpitation, had been found by most authors [7,8]. It is a circumstance of discovery of secreting forms of pheochromocytomas and paragangliomas [7]. Our patient did not present a typical clinical table. His main symptom was right hypochondrium pain which can be confused with tumours of body of the pancreas. Despite the presence of a known and isolated hypertension, the absence of this triad led to preoperative misdiagnosis and contempt for diagnosis. Biology is an important step in diagnosing pheochromocytomas and paragangliomas [1,[9], [10], [11]]. The dosage of metanephrines and normetanephrines enables preoperative diagnosis and a better preparation of surgery [1,[9], [10], [11]].

Imaging helps localize the tumour and give its features. It holds an important place in the diagnosis of pheochromocytomas and paragagliomas [2,10,12,13]. In our case, CT scan was the diagnostic examination, but did not contribute to an accurate diagnosis of the organ. Magnetic Resonance Imaging (MRI), MIBG scintigraphy, and PET-Scan contribute to diagnostic accuracy [2,14].

Preoperative medical preparation is aimed at reducing risks of peroperative hemodynamic instability [15,16]. Preoperative hypertension peaks up to 250 mmHg were reported [17,18]. Besides, it is the rise in blood pressure upon contact with the tumour that helped adjust our diagnosis. The anesthetist should therefore prepare himself to manage blood pressure peaks during the tumour’s dissection, but also the possible low blood pressure at the end of exeresis. Surgery remains the key element of treatment and consists in exeresis of the paragaglioma. Our patient had a laparotomy. However laparoscopy allows resection with fewer scars and postoperative complications [20]. Anatomopathological analysis of the excised tissues provides an accurate diagnosis of pheochromocytoma [1].

In the literature, there is almost no mortality, thanks to the progress of medical imaging and biology, which allow preoperative diagnosis [4,19].

## 4 Conclusion

Pheochromocytoma is a rare tumor. The Menard Triad which is the typical clinical observatory. This explains why the diagnosis was an operative surprise. A preoperative diagnosis based on clinical, radiological and biological arguments would help better reorganize resuscitation, the only guarantee of good prognosis. It is therefore important to publish this case in the English literature.

## Declaration of Competing Interest

The authors declare that they have no competing interests regarding the publication of this manuscript.

## Sources of funding

No sponsors to declare.

## Ethical approval

Ethical approval is not needed for this case report as patient consent and we are not trialing a new device.

## Consent

Written and signed consent by the patient to publish a case report has been obtained.

## Author contribution

Case report concept and design: Zongo N, KOAMA A, Kambou/Tiemtoré B.

Acquisition of data: KOAMA A, Zongo N, Nde/ Ouédraogo NA, LOMPO OM.

Statistical analysis and interpretation of data: KOAMA A, Sanou A, Diallo O, Lougué /Sorgho C, Cissé R.

Drafting of the manuscript: KOAMA A, Sanou A, Lougué /Sorgho C, Cissé R, HOURY S.

Critcal revision of the manuscript for important intellectual content: KOAMA A, Zida M, Ouédraogo MNL, Ouangré E, Sanou A, Lompo OM, Sanou A, Diallo O, Lougué /Sorgho C, Cissé R.

All authors approved the final version of this publication.

## Registration of research studies

It is not a clinical trial.

## Guarantor

Dr Nayi Zongo.

## Provenance and peer review

Not commissioned, externally peer-reviewed.
